# Wsv023 interacted with *Litopenaeus vannamei* γ-tubulin complex associated proteins 2, and decreased the formation of microtubules

**DOI:** 10.1098/rsos.160379

**Published:** 2017-04-26

**Authors:** Yihong Chen, Haitao Bi, Xiaoyun Li, Zezhi Zhang, Haitao Yue, Shaoping Weng, Jianguo He

**Affiliations:** 1 Guangdong Provincial Key Laboratory of Marine Resources and Coastal Engineering, South China Sea Bio-Resource Exploitation and Protection Collaborative Innovation Center (SCS-REPIC), School of Marine Sciences, Sun Yat-sen University, 135 Xingang Road West, Guangzhou 510275, People's Republic of China; 2 School of Life Sciences, Sun Yat-sen University, 135 Xingang Road West, Guangzhou 510275, People's Republic of China; 3 State Key Laboratory for Biocontrol, OE Key Laboratory of Aquatic Product Safety, Institute of Aquatic Economic Animals and Guangdong Province Key Laboratory for Aquatic Economic Animals, Sun Yat-sen University, 135 Xingang Road West, Guangzhou 510275, People's Republic of China; 4 Fisheries College, Guangdong Ocean University, Zhanjiang, People's Republic of China

**Keywords:** unfolded protein response, *Litopenaeus vannamei*, white spot syndrome virus, wsv023

## Abstract

A previous study found the key transcription factor of *Litopenaeus vannamei* PERK-eIF2α pathway cyclic AMP-dependent transcription factor 4 (LvATF4) was involved in the transcriptional regulation of white spot syndrome virus (WSSV) gene *wsv023*. Knocked-down expression of LvATF4 reduced the viral copy number and the cumulative mortality of WSSV-infected shrimp. These results suggested that wsv023 may be critical to WSSV infection but the precise function of wsv023 was still unknown. By using co-immunoprecipitation and pull-down assays, we show that wsv023 interacts with *L. vannamei* gamma complex-associated protein 2 (LvGCP2), which is the core protein of the γ-tubulin small complex. Knocked-down, the *wsv023* gene significantly reduced the copy number of WSSV in *L. vannamei* muscle, as well as the cumulative mortality of infected shrimp. And PERK-eIF2α pathway inhibition also showed reduced virus copy number and abrogated shrimp mortality. Furthermore, overexpression of wsv023 inhibited the formation of microtubules in 293T cells. Flow cytometry revealed that WSSV infection similarly decreased the formation of microtubules in *L. vannamei* haemocytes. These findings suggested that wsv023 plays a role in microtubule organization in host cells, which in turn may be beneficial to WSSV.

## 1. Introduction

Microtubules are essential components of the cytoskeleton that confers shape and function in eukaryotic cells. They also play a vital role in intracellular material transportation, organelle positioning, cell movement, signal transduction and cell division [1]. Microtubules should be ideally assembled using a special mode that is convenient for rapid reorganization and decomposition [2]. Research studies have shown that microtubules are formed by the arrangement of 13 parallel protofilaments that arise from the end-to-end aggregation of the α/β tubulin-dimers [3]. Microtubules are associated with various other proteins, such as dynein and kinesin, which are involved in the organization of the microtubule cytoskeleton [4].

Cells have specialized nucleation sites that are found in microtubule-organizing centres (MTOCs) [5]. All MTOCs contain γ-tubulin, which is homologous to α-tubulin and β-tubulin and is required for microtubule nucleation [6]. γ-tubulin is prevalent throughout the eukaryotes and is essential for normal microtubule organization in every organism. However, γ-tubulin is involved in nucleation of cells in non-MTOC sites, such as plant cells, which lack centrosome-like structures [7]. Thus, γ-tubulin seems to be critical to the initiation of new microtubules *in vivo*. γ-tubulin is part of a large complex consisting of several proteins, such as γ-tubulin, which is purified from *Drosophila melanogaster* embryos or *Xenopus laevis* eggs. This complex contains γ-tubulin complex protein (GCP) 2, GCP3, GCP4, GCP5, GCP6 and the neural precursor cell expressed developmentally downregulated protein 1 (NEDD1) that form a γ-tubulin ring (γ-TuRC). In γ-TuRC, γ-tubulin interacts with GCP2 and GCP3 and yields a stable 300 kDa subcomplex, called γ-tubulin smallcomplex (γ-TuSC) [8]. The assembly state of the γ-tubulin complex is important for its activity, and purified γ-TuSC has much lower microtubule-nucleating activity than intact γ-TuRC [3].

A previous investigation has determined that viral infections might influence microtubule organization in host cells. In the neuroblastoma cell line IMR32, a microtubule network was used as the transport system of a virus replication site and is involved in the release of mature Japanese encephalitis viruses [9]. A 60% decrease in particulate tubulin synthesis was reported in SV403T3 cells after transformation by SV40 virus compared to that in normal cells [10]. The matrix protein of the vesicular stomatitis virus interacted with tubulin *in vivo* and *in vitro*, suggesting that a cytopathic effect was caused by these interactions [11]. In herpes simplex virus-1 (HSV-1) infection disperses the location of miniaturized MTs in the cytoplasm, which in turn facilitates HSV-1 capsid migration to the site of envelopment after exiting the nucleus, thereby promoting efficient viral spread [12].

White spot syndrome virus (WSSV) is a rapidly replicating and extremely virulent shrimp pathogen that causes huge economic losses in shrimp aquaculture. Some studies have indicated that cytoskeleton modulation in shrimp cells might be linked to WSSV infection-driven microtubule modifications [13–16]. However, the relationship between WSSV infection and host cell microtubule modification remains unclear. This study proved that WSSV gene wsv023 interacted with *Litopenaeus vannamei* GCP2 (LvGCP2), the core member of γ-TuSC. Overexpression of wsv023 in 293T cells inhibited the formation of microtubules. Silencing expression of *wsv023* significantly reduced the WSSV copy number in *L. vannamei* muscle and the cumulative mortality of WSSV-infected shrimp. These results suggest that WSSV possibly affects microtubule organization in the host cells via wsv023, thereby enhancing the infection process.

## 2. Material and methods

### 2.1. Co-immunoprecipitation and western blot assay

Expression vectors for full-length LvGCP2, eGFP with Flag-tag or wsv023, and GST with V5-tag were constructed for co-immunoprecipitation (Co-IP) assays. Briefly, *Drosophila* S2 cells were co-transfected with the expression plasmid pairs LvGCP2 (Flag)/wsv023 (V5), LvGCP2 (Flag)/GST(V5) and eGFP (Flag)/wsv023 (V5). Cells were harvested at 48 h post-transfection. S2 cells were lysed in NP-40 lysis buffer (Beyotime, China) on ice with a protease inhibitor cocktail (Roche Applied Science, Germany) and then incubated for 2 h at 4°C with anti-V5 or anti-Flag antibodies (Invitrogen, USA). The proteins were immunoprecipitated with Protein G Plus/protein A Agarose (Calbiochem, Germany). After incubation, the beads were washed three times with NP-40 lysis buffer, subjected to SDS-PAGE, and then electrotransferred to nitrocellulose membranes. Then western blot analyses were then performed. The blots were incubated for 2 h at room temperature (RT) in tris buffered saline with Tween-20 (TBST) with 5% bovine serum albumin and then for 1.5 h at RT with the anti-Flag or anti-V5 antibody. After washing three times with TBST, the membranes were incubated in TBST with 1/20 000 horseradish peroxidase (HRP)-conjugated goat anti-mouse IgG antibody (Pierce, USA) for 1 h at RT. Signals were detected with Superstar ECL Plus (Pierce).

### 2.2. Pull-down assay

Prokaryotic expression vectors for N-terminal LvGCP2 (1 ∼ 450 aa, LvGCP2N) and the full-length open reading frame (ORF) of the *wsv023* gene were constructed (for information on the primers used in the study, see table 1) by inserting the corresponding DNA fragments into pMAL-c2X (New England BioLabs, Inc.) and pET32a^+^ (Novergen, USA), respectively. The recombinant proteins were expressed in *Escherichia coli* (*BL21*). The supernatants contained recombinant proteins, which were mixed together in the following pairs: MBP-LvGCP2N and 6×His-wsv023, MBP-LvGCP2N and 6×His-GST, and MBP-eGFP and 6×His-wsv023; each of these pairs were then incubated at 4°C in a vertical mixer for 4 h. The MBP and MBP-LvGCP2N were purified with amylose beads (New England BioLabs, Inc.), and 6×His-GST and 6×His-wsv023 were purified with Ni-NTA Agarose (Qiagen, USA), all according to the respective manufacturers’ instructions. The beads were then collected, extensively washed and boiled in a sample buffer, followed by SDS-PAGE and then western blot assays.

**Table 1.** Summary of primers used in this study.

primers	sequence (5′–3′)
*for cDNA cloning*	
LvGCP2-5race1	CGTGTGATAAGGCCGGTTATCCAGCCAA
LvGCP2-5race2	TTTCTCCTCTTTCAACTGCTCGCATGGC
LvGCP2-3race1	ACCTGCTGAGCCTCATGATGGGGATAGA
LvGCP2-3race2	ATGACGCCCCAGAGTTTACTGTCGATCC
LvGCP3-5race1	ATCTGAAGCAATGAAGAACTCGTGGTGA
LvGCP3-5race2	GAGCATAACATACAGAGGTGTGCAGGCC
LvGCP3-3race1	ACCTGTACCCACATAATCTGTCTTCCCT
LvGCP3-3race2	CTCAGTATGAGGAACCAGACATCCTCGA
	
*for gene expression*^a^	
pACB-LvGCP2-KpnI-Flag-F	ATA**GGTACC**ATGGATTACAAGGATGACGACAGTGAGTTTAAAATCCACCACC
pACB-LvGCP2-Xba I-R	ATATCTAGACTTCACCCTGAAACCGGGCCGCTG
pACB/IZ-wsv023-EcoR I-F	ATA**GAATTC**TATGAGCTCGGGTAGTATCAACAA
pACB/IZ-wsv023-CG-Xho I-R	TTA**CTCGAG**CGTAATTTCTCAGAAAAGTTCCTCAAC
pCDNA3-Flag-wsv023-F1	ATGGATTACAAGGATGACGACGATAAGATGAGCTCGGGTAGTATCAACA
pCDNA3-Flag-wsv023-BamH I-F2	TAT**GGATCC**ATGGATTACAAGGATGACGACGATAAG
pCDNA3-Flag-wsv023-Xho I-R	TTA**CTCGAG**TCATAATTTCTCAGAAAAGTTCCTC
pCDNA3-Flag-wsv220(N250)-F1	ATGGATTACAAGGATGACGACGATAAGATGGCAGGGAATAGAACCC
pCDNA3-Flag-wsv220(N250)-BamH I-F2	TAT**GGATCC**ATGGATTACAAGGATGACGACGATAAG
pCDNA3-Flag- wsv220(N250)-Xho I-R	TTA**CTCGAG**CTACGTCCATTTTGGTGGAAAGATA
pACB-LvGST-Kpn I-F	ATA**GGTACC**AAAATGGCCCCTATACTAGGTTATTGG
pACB-LvGST-Apa I-R	TAT**GGGCCC**TCGTCACGATGCGGCCGCTCGA
pACB/IZ-eGFP-Kpn I-Flag-F1	ATA**GGTACC**ATGGATTACAAGGATGACGACGTGAGCAAGGGCGAGGAGCT
pACB/IZ-eGFP-EcoR I-Flag-R	TAT**GAATTC**CACTACTTATCGTCGTCATCCTTGTAATC
pET32A-wsv023-Kpn I-F	TTA**GGTACC**ATGAGCTCGGGTAGTATCAACA
pET32A-wsv023-BamH I-R	ATT**GGATCC**TCATAATTTCTCAGAAAAGTTCCTCAA
pET32A-eGFP-Kpn I-F	TTA**GGTACC**ATGGTGAGCAAGGGCGAGG
pET32A-eGFP-BamH I-R	ATT**GGATCC**CTTGTACAGCTCGTCCATGCC
pMAL-C2X-LvGCP2-BamH I-450-F	AAT**GGATCC**ATGAGTGAGTTTAAAATCCACCAC
pMAL-C2X-LvGCP2-Sal I-450-R	ATA**GTCGAC**TCATTTCCCAGTTCGCAGAATGA
	
*for dsRNA template amplification*	
DsRNA-wsv023-469-T7-F1	GGATCCTAATACGACTCACTATAGGTTTGGAGAATCTGCATCAGAGA
DsRNA-wsv023-469-R1	GAATTGCAGTGATAGGCACAG
DsRNA-wsv023-469-F2	TTTGGAGAATCTGCATCAGAGA
DsRNA-wsv023-469-T7-R2	GGATCCTAATACGACTCACTATAGGGAATTGCAGTGATAGGCACAG
DsRNA-eGFP504-T7-F1	GGATCCTAATACGACTCACTATAGGCGACGTAAACGGCCACAAGTT
DsRNA-eGFP504-R1	ATGGGGGTGTTCTGCTGGTAG
DsRNA-eGFP504-F2	CGACGTAAACGGCCACAAGTT
DsRNA-eGFP504-T7-R2	GGATCCTAATACGACTCACTATAGGATGGGGGTGTTCTGCTGGTAG
*for RT-PCR*	
SQPCR-wsv023-536-F	GAAGTTGACTCCATTATTCGCCA
SQPCR-wsv023-536-R	AAGTTCCTCAACAGGGGAATTATAG
SQ-PCR-LvEF1α-329-F	CCAGGGTGAAGCACAGCAAC
SQ-PCR-LvEF1α-329-R	CGACAAGCGAACCATCGAGA
	
*for real-time RT-PCR*	
QPCR-Lvα-Tubulin-F	CGTCGAGCCCTACAACTCCAT
QPCR-Lvα-Tubulin-R	GCCTCGTTGTCGACCATGA
QPCR-Lvβ-Tubulin-F	TCCAAGATCCGGGAGGAGTA
QPCR-Lvβ-Tubulin-R	CGGTATGACGGAGAACGTGTT
QPCR-Lvγ-Tubulin-F	ACGTAATGCGTCGCTTGCT
QPCR-Lvγ-Tubulin-R	CGATCCAAGGGAGTAGAGACCAT
QPCR-wsv069-F	TGTTTTCTGTATGTAATGCGTGTAGGT
QPCR-wsv069-R	CCCACTCCATGGCCTTCA
QPCR-LvEF-1α-F	AAGGCCCTCAAGAAGAAGTAAAT
QPCR-LvEF-1α-R	TTGACAACCATACCTGGCTTC

^a^Nucleotides in bold indicate restriction sites introduced for cloning.

### 2.3. Bioinformatics analysis

The GCP2 or GCP3 protein sequences from other species in the database were searched and analysed using the BLAST program (http://www.ncbi.nlm.nih.gov/BLAST/). Multiple sequence alignment was performed using the ClustalW program. Using MEGA 6.0 software, a neighbour-joining (NJ) phylogenetic tree was constructed based on the deduced amino acid sequences of LvGCP2 and other known GCP2 proteins. Bootstrap sampling was performed 10 000 times. Protein domains were predicted using the SMART program (http://smart.embl-heidelberg.de/).

### 2.4. Flow cytometry assay

293T cells were transfected with plasmids pcDNA-wsv023 and pcDNA-wsv220N250 (negative control, 1 ∼ 250 aa of WSSV membrane protein wsv220). After 48 h, the quantity of microtubules was examined using Tubulin Tracker™ Green kit (Life Technology, USA) following the manufacturer's instructions. A sufficient amount of staining solution (250 nM) was applied to cover the cells and then incubated for 30 min at 37°C. After incubation, the staining solution was removed, and the cells were rinsed three times in a buffer at 37°C. Then the cells were gently resuspended with Hank's balanced salt solution (HBSS) gently. Approximately 5 × 10^5^ cells were applied to flow cytometry assay by BD accuri system (BD Biosciences, USA), and the fluorescence value of the cells were autokinetic calculated by BD accuri C6. Total RNAs and proteins of 293T cells from different groups were extracted, and the expression of *Homo sapiens* α-tubulin, β-tubulin and β-actin was detected by real-time RT-PCR and western blot. In the WSSV infection assay, haemocytes were collected from five shrimps in the phosphate buffered saline (PBS) injection group and the WSSV infection group at 72 h after treatment; the haemocytes were pooled together as one sample (*n* = 3). *Litopenaeus vannamei* haemocytes were prepared as described elsewhere [17].

### 2.5. Synthesis of double-stranded RNA

The DNA templates of the *wsv023* gene for dsRNA- (designated as dsRNA-wsv023) synthesis were prepared via PCR using the primer pairs DsRNA-wsv023-T7-F/DsRNA-wsv023-R and DsRNA-wsv023-F/DsRNA-wsv023-T7-R (table 1). The DNA template for ssRNA synthesis consisted of annealed DNA oligonucleotides containing a T7 RNA polymerase promoter. For sense ssRNA synthesis, a DNA template containing a T7 RNA polymerase promoter at its 5′-end was employed, so the T7 RNA polymerase promoter sequence was added to 5′-end of the forward primer. For antisense ssRNA synthesis, the DNA template contained a T7 RNA polymerase promoter at its 3′-end, so a T7 RNA polymerase promoter sequence was added to the 5′-end of the reverse primer. The products with the T7 promoter were confirmed by sequencing, and then subjected to *in vitro* transcription with the RiboMAX large-scale RNA production (Promega, USA) according to the instruction manual. The templates of the eGFP dsRNA (designated as dsRNA-eGFP) were prepared via PCR using the primer pairs DsRNA-eGFP-T7-F/DsRNA-eGFP-R and DsRNA-eGFP-F/DsRNA-eGFP-T7-R (table 1). The lengths of dsRNA-wsv023 and dsRNA-eGFP were 469 and 504 bp, respectively.

### 2.6. White spot syndrome virus challenge and preparation of templates for real-time RT-PCR analysis

To investigate the expression profiles of *Lvα-tubulin*, *Lvβ-tubulin* and *Lvγ-tubulin* in WSSV-infected shrimp, WSSV challenge was performed. Each healthy *L. vannamei* was injected intramuscularly at the second abdominal segment with 50 µl of a WSSV inoculum, which contained approximately 10^4^ virions. Total RNA from the haemocytes was isolated at 0, 3, 6, 9,12, 18, 24, 30, 36, 48, 72 and 96 h post inoculation (hpi). From each sample, total RNA was extracted using RNeasy Mini Kit (Qiagen, Germany) and then reverse transcribed into cDNA by using PrimeScript RT Reagent Kit (TaKaRa, Japan). Then, real-time RT-PCR assay was performed on a LightCycler 480 System (Roche, Germany) using a reaction volume of 10 ml, which comprised 1 ml of cDNA template, 5 ml of 2× SYBR Premix Ex Taq™ II (Takara), and 500 nM of each primer. The optimized thermal cycling parameters were as follows: 95°C for 2 min to activate the polymerase, followed by 40 cycles at 95°C for 15 s, 60°C for 15 s and 72°C for 10 s. After performing the cycling protocol, the melting curves were obtained by increasing the temperature from 72°C to 95°C (0.5°C s^−1^) to denature the double-stranded DNA. The results were calculated using the 2^−ΔΔ*Ct*^ method after normalization to *LvEF-1a* (GenBank Accession No. GU136229) [18]. The primer sequences are presented in table 1.

### 2.7. Bioassay of white spot syndrome virus challenge tests in *wsv023* knocked-down shrimp

Juvenile shrimp of 6 g mean body weight were obtained from the south base of a marine culture seed project (Zhanjiang, China) and screened for WSSV by PCR using WSSV-specific primers (5′-TCGCCATCACTGCTGTGATTGC-3′ and 5′-CTTTGGCACCATC-TGCATACC-3′). In cumulative mortality assay, for each treatment, 50 × 2 shrimp were intramuscularly injected with 6 µg (50 µl in volume) of dswsv023, dseGFP, or PBS, respectively; 48 h later, 50 shrimp of each treatment were injected with 50 µl of PBS (negative controls), and another 50 shrimp were injected with tissue homogenate containing 10^4^ virions (50 µl in volume) that was prepared from WSSV-infected shrimp following a published protocol [19]. The experiment was repeated three times. All shrimp were kept in culture tanks for approximately 7 days following infection. Cumulative mortality was recorded every 12 h.

To determine WSSV copy number, at each time point (a total of four time points), the muscles of five shrimp were collected and pooled into one sample. Three parallel samples were prepared. Then WSSV copy number was determined by real-time RT-PCR.

At 48 h after dseGFP or dswsv023 injection, a subsequent injection of WSSV-infected tissue homogenate was performed to examine the RNAi efficiency of *wsv023*, and shrimp were sacrificed on the third day after WSSV injection. Total RNA that was extracted from the haemocytes was reverse transcribed into cDNA using the PrimeScript RT reagent kit (TaKaRa) and then subsequently used as templates. *LvEF1α* was used as the internal reference. The primer sequences used in this assay are listed in table 1.

### 2.8. Bioassay of white spot syndrome virus challenge tests in PERK-eIF2α pathway inhibited shrimp

Salubrinal is a selective inhibitor of eIF2α dephosphorylation that inhibits endoplasmic reticulum-stress in cells. In a previous study, we showed that *wsv023* is upregulated by the PERK-eIF2α pathway in *L. vannamei* [20]. In this study, shrimp (*n* = 100) were injected with Salubrinal (20 µM) or 0.1% dimethyl sulphoxide (DMSO). One hour later for each treatment, 50 shrimp were injected with tissue homogenate containing 1 × 10^4^ virions (50 µl in volume), and 50 shrimp were injected with PBS. Cumulative mortality was recorded every 12 h. For the western blot assay and WSSV virion detection, an additional 120 shrimp were prepared with salubrinal (20 µM) or 0.1% DMSO injection plus WSSV infection.

## 3. Results

### 3.1. Wsv023 interaction with LvGCP2

**Figure 1.**
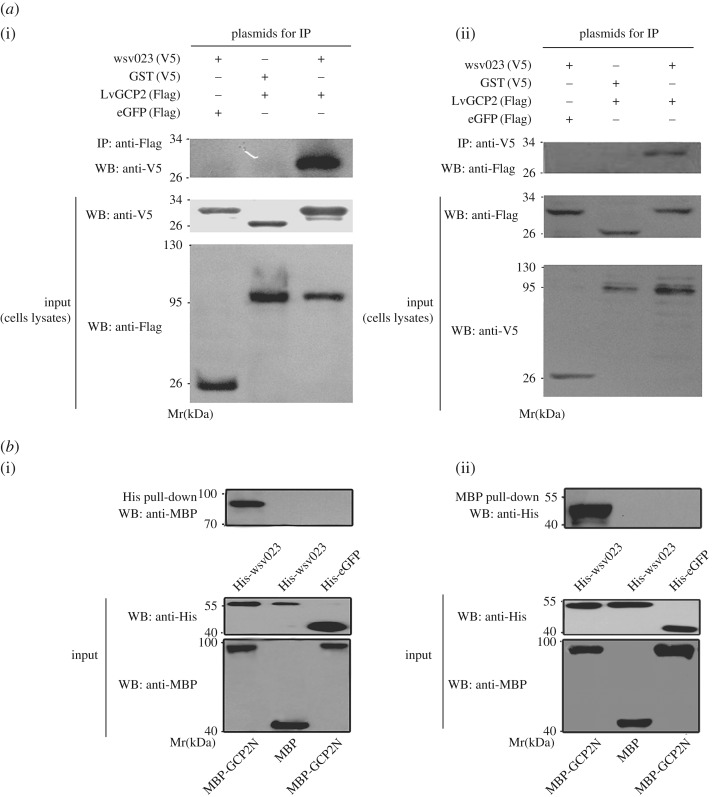
Interactions between w023 and LvGCP2. (*a*) Co-IP assay revealed interactions between LvGCP2 (FLAG) and wsv023 (V5); (*b*) pull-down assay confirmed interactions between w023 and LvGCP2. The blots were resolved by SDS-PAGE and visualized by immunoblotting with anti-V5 or anti-Flag antibody.

In our previous study, tandem affinity purification indicated that wsv023 probably interacted with LvGCP2. To verify whether wsv023 formed a complex with LvGCP2 in *L. vannamei*, co-IP and pull-down assays were conducted. The Wsv023 (V5) and LvGCP2 (Flag) proteins were co-expressed in S2 cells for the co-IP, which showed that wsv023 (V5) and LvGCP2 (Flag) co-precipitated with each other (figure 1*a*). Furthermore, the MBP pull-down assay showed that MBP-LvGCP2N interacted with 6×His-wsv023 but not with 6×His-eGFP (V5) (figure 1*b*). In addition, MBP alone did not interact with wsv023 (figure 1*b*). These findings thus indicated that wsv023 interacted with LvGCP2.

### 3.2. Characterization of LvGCP2 and wsv023

The full-length cDNA of *LvGCP2* was 3155 bp in size, which included an 88-bp 5′-untranslated region (UTR) and a 526-bp 3′-UTR with a poly(A) tail (electronic supplementary material, figure S1). The ORF of *LvGCP2* was 2541 bp in size, which encodes a putative protein of 846 amino acids with a calculated molecular weight of 97 kDa. Conserved domain analysis using the SMART program showed that LvGCP2 contained a Spc97_Spc98 domain that apparently mainly consists of proteins that functioned in microtubule cytoskeleton organization. Multiple sequence alignment showed that the GCP2 proteins were conserved in all species tested (electronic supplementary material, figure S2). Furthermore, LvGCP2 showed a higher identity with vertebrate GCP2s than most of the invertebrate GCP2 proteins except for ZnGCP2; this result coincided with the results of phylogenetic analysis (electronic supplementary material, figure S1).

Wsv023 did not show any conserved domain as analysed by SMART (http://smart.embl-heidelberg.de/), whereas BLAST analysis (http://blast.ncbi.nlm.nih.gov/Blast.cgi) revealed that wsv023 has similarities with GCP3s. Multiple sequence comparisons indicated that wsv023 had 20.6% and 16.0% identities with *H. sapiens* GCP3 (HsGCP3) and LvGCP3, respectively (electronic supplementary material figure S3). Considering the amino acids with similar properties, wsv023 showed 35.2% and 31.3% identity with HsGCP3 and LvGCP3, respectively (electronic supplementary material figure S3).

### 3.3. Knocked-down expression of *wsv023* decreased shrimp cumulative mortality and copy number of white spot syndrome virus in muscle of white spot syndrome virus-infected *Litopenaeus vannamei*

**Figure 2.**
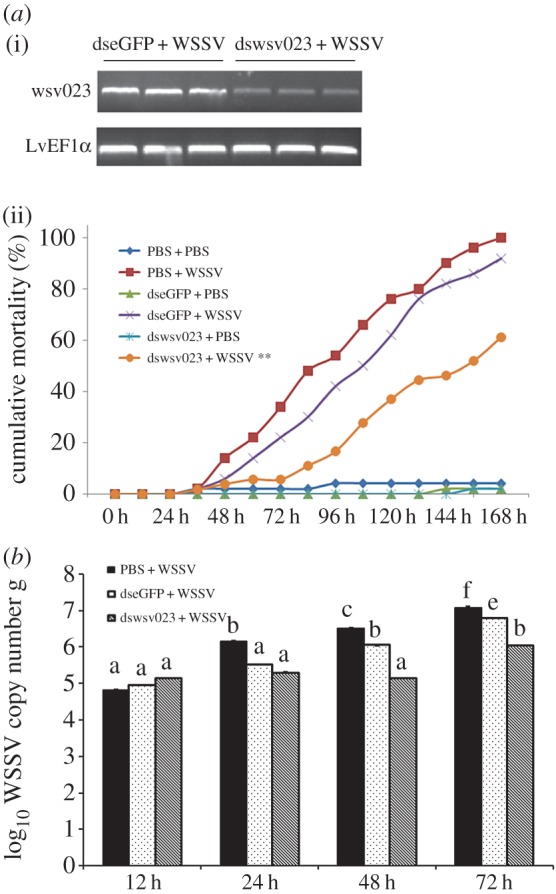
Cumulative mortality or WSSV copy number decreased in shrimps treatment with dswsv023 plus WSSV infection. RT-PCR analysis gene expression of wsv023 (*a*(i)), all samples were taken 72 h after injection with indicated dsRNA. The internal control was *LvEF1α*. (*a*(ii)) Shrimp (*n* = 50) were injected intramuscularly with PBS, dswsv023 or dseGFP. At 48 h after the initial injection, shrimps were infected with WSSV or PBS again (negative control), and cumulative mortality of shrimps was recorded every 12 h. (*b*) The bars indicate mean values ± s.d. of the log_10_ WSSV copy number per 1 g muscle (*n* = 3). The statistical significance was calculated using Student's *t*-test (** indicates *p* < 0.01 compared with control).

Treatment with dsRNA-wsv023 on WSSV-infected shrimp effectively suppressed its expression in *L. vannamei* haemocytes as shown by RT-PCR assay (figure 2*a*(i)). The shrimp treated with dsRNA-wsv023 had low cumulative mortality 72 h post WSSV infection. Shrimps treated with dsRNA-wsv023 and challenged with WSSV had a cumulative mortality of 60% at 168 hpi. By contrast, groups mock treated with dsRNA-eGFP or PBS had cumulative mortalities of 92% and 100% at 168 hpi, respectively (figure 2*a*(ii)).

The WSSV copy number in the *wsv023* gene knocked-down groups was examined at 12, 24, 48 and 72 h after WSSV challenge (electronic supplementary material, table S1). At 12 hpi, the WSSV copy number in *L. vannamei* muscles showed no significant differences between the *wsv023* knocked-down groups and the control groups (figure 2*b*). At 24 hpi, the copy number of *wsv023* knocked-down groups showed significantly lower copy numbers than that in the PBS injection groups, except for the dsRNA-eGFP injection group. At 48 and 72 hpi, *wsv023* knocked-down groups had significantly lower WSSV copy number than the control groups (figure 2*b*). At 72 hpi, WSSV copy number in the PBS injection group was about 10-fold higher than that of the wsv023 knocked-down group, and at 48 and 72 hpi, WSSV copy number in dsRNA-eGFP treated groups was significantly higher than the *wsv023* knocked-down groups too (figure 2*B*).

### 3.4. PERK-eIF2α pathway inhibition decreased white spot syndrome virus infection in shrimps

**Figure 3.**
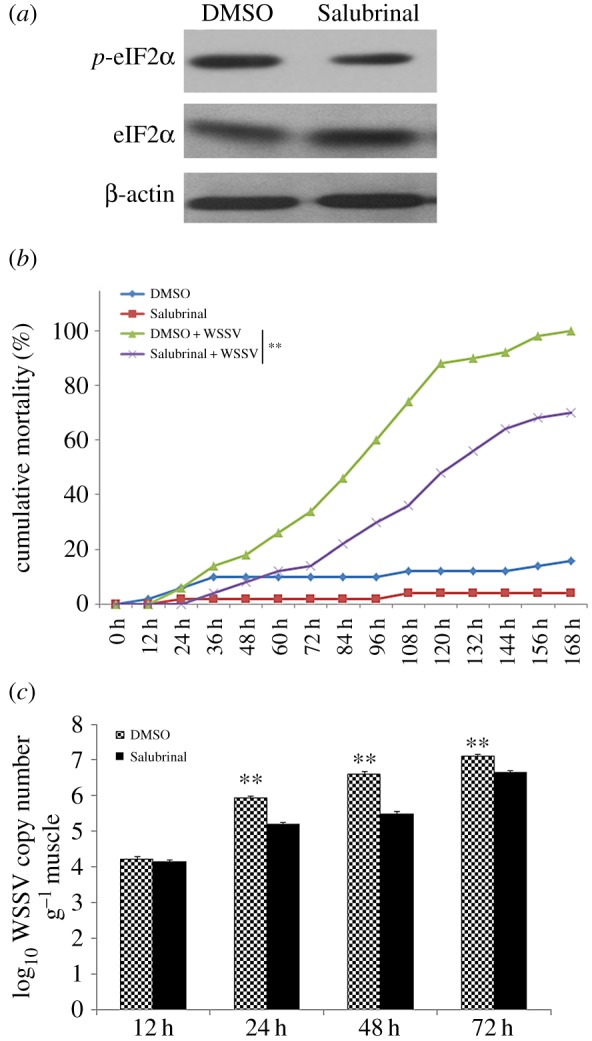
PERK-eIF2α pathway inhibition restrained WSSV infection. Shrimp were injected with Salubrinal or DMSO (negative control). (*a*) Western blot assay showed that Salubrinal injection reduced the phosphorylation of eIF2α compared with the control; (*b*) cumulative mortality was recorded every 12 h post-challenge. Data were derived from three independent experiments and shown as mean ± s.d. Differences in mortality levels between treatments were analysed by Kaplan–Meier plot (log-rank *Х*^2^ test). Significant differences in *L. vannamei* mortality were marked with asterisks (** indicates *p* < 0.01); (*c*) DNA were collected from shrimp and detected WSSV copy numbers in each samples by quantity RT-PCR. Data were expressed as mean fold change (mean ± s.d., *n* = 3) from the untreated group, significant differences in *L. vannamei* mortality were marked with asterisks (** indicates *p* < 0.01).

Western blot assay showed that 48 h after Salubrinal injection, phosphorylation of eIF2α was reduced in haemocytes of *L. vannamei* (figure 3*a*). At 24, 48 and 72 hpi, the WSSV copy number in *L. vannamei* muscle of the Salubrinal treatment group was significantly lower than that of the respective control groups (figure 3*c*). The shrimps treated with Salubrinal also had lower cumulative mortality than the control groups (figure 3*b*). The cumulative mortalities of the DMSO injection group, Salubrinal injection group, DMSO injection plus WSSV infection group and Salubrinal injection plus WSSV infection group at 168 hpi were 16%, 4%, 100% and 70%, respectively (figure 3*b*).

### 3.5. Wsv023 inhibited tubulin polymerization in 293T cells

**Figure 4.**
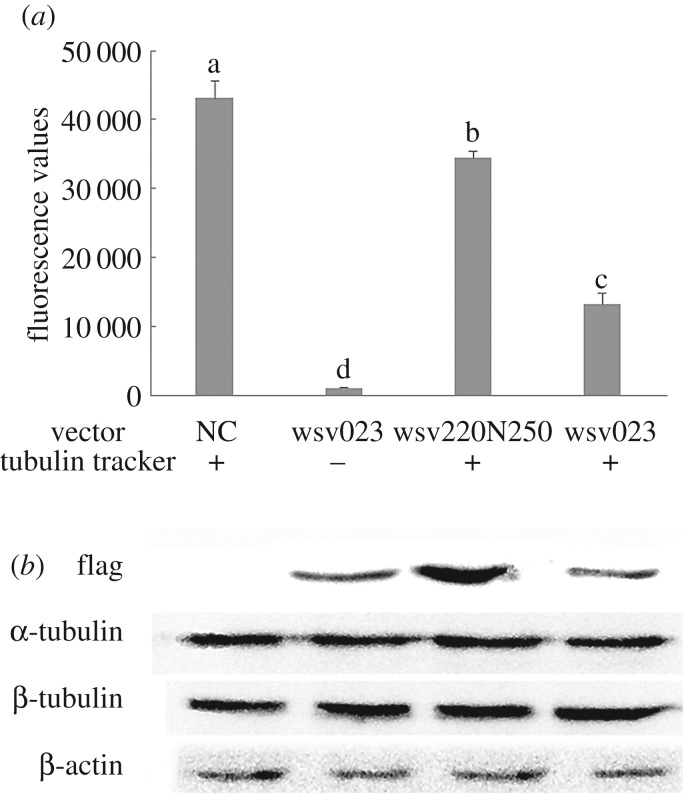
Overexpressed *wsv023* in 293T cells decreased the microtubules formation. (*a*) Cells overexpressed wsv023 with fluorescence value significant lower that overexpressed wsv220N250, with tubulin tracker treatment. (*b*) Cells overexpressed wsv023 did not decrease the expression of α/β-tubulins. Data were expressed as mean fold change (mean ± s.d., *n* = 3) from the untreated group. The bars with different letters indicate statistical differences (*p* < 0.05). Statistical significance was determined by one-way ANOVA.

TubulinTracker™ Green reagent (T34075) provides green fluorescent staining of polymerized tubulins, but not the unpolymerized tubulins in live cells. Wsv023 was overexpressed in 293T cells to investigate the function of wsv023. Flow cytometry analysis showed that wsv023 significantly decreased the average fluorescence values of the 293T cells, which was 20% less than that of the wsv220N250 expression groups (figure 4*a*). Western blot analysis showed that wsv023 did not affect the expression of α-tubulin and β-tubulin (figure 4*b*). Thus, the reduction in tubulin polymerization in 293T cells that overexpressed wsv023 was not influenced by the supply of α/β-tubulin. Given that γ-TuSC is critical for initiating all new microtubules [3], the present results suggested that wsv023 interacted with LvGCP2, and might therefore influence microtubule formation.

### 3.6. WSSV infection upregulates *Lvα-tubulin* and *Lvβ-tubulin* and inhibits microtubule formation

**Figure 5.**
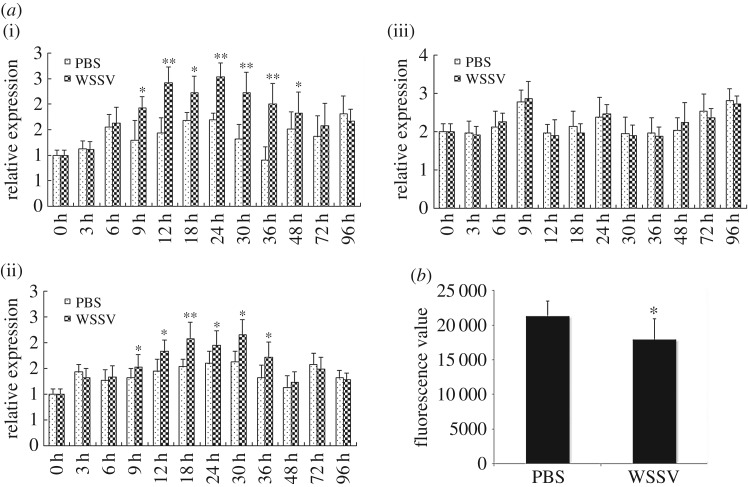
Expression profile of *Lvα-tubulin*, *Lvβ-tubulin* and *Lvγ-tubulin*, and microtubules formation condition in haemocytes of WSSV-challenged *L. vannamei*. The mRNAs were collected at 0, 3, 6, 9, 12, 18, 24, 30, 36, 48, 72 and 96 hpi. The expression level of *Lvα-tubulin*, *Lvβ-tubulin* and *Lvγ-tubulin* was measured by real-time RT-PCR. The relative expression of *Lvα-tubulin* (*a*(i)), *Lvβ-tubulin* (*a*(ii)) and *Lvγ-tubulin* (*a*(iii)) after injection with the WSSV in haemocytes were normalized to *LvEF-1a* and compared against time zero. The results were based on three independent experiments and expressed as the mean values ± s.d. Statistical significance was determined by Student's *t*-test (*indicates *p* < 0.05, **indicates *p* < 0.01 compared with controls at each time point). (*b*) Haemocytes collected from WSSV-infected shrimp with fluorescence value significant lower than the control group.

cDNA microarray analysis indicated that WSSV infection affects the expression of several genes that are relevant to microtubule formation [21]. *Lvα-tubulin*, *Lvβ-tubulin* and *Lvγ-tubulin* are primary components of *L. vannamei* microtubules. In the present study, RT-PCR analysis revealed that the expression of *Lvα-tubulin* (figure 5*a*(i)) and *Lvβ-tubulin* (figure 5*a*(ii)) in the haemocytes was upregulated after WSSV injection, whereas that of *Lvγ-Tubulin* (figure 5*a*(iii)) was not. The relative expression levels of *Lvα-Tubulin* and *Lvβ-Tubulin* reached peak values at 24 and 30 hpi and were approximately 1.5- and 1.3-fold that of the control, respectively. Flow cytometry assay indicated that WSSV infection significantly reduced microtubule formation in shrimp *L. vannamei* haemocytes by about 16% at 72 hpi (figure 5*b*).

## 4. Discussion

Growing evidence indicates that WSSV infection modifies microtubule organization in host cells, yet more research is needed to make it clear. Blast analysis showed that wsv023 was similar to the GCP3 sequence of species such as *H. sapiens*. Other assays (Co-IP and pull-down) proved that wsv023 could interact with the core protein GCP2 of γ-TuSC in *L. vannamei*. Further, silencing *wsv023* reduced WSSV copy number in muscle and cumulative mortality of WSSV-infected shrimp. Moreover, flow cytometry analysis revealed that wsv023 might play a role in inhibiting microtubule formation in host cells. These results indicate that *wsv023* influences microtubule synthesis and organization in WSSV-infected cells in shrimp.

A total of 684, 531 and 507 putative ORFs have been identified on the WSSV-Th, WSSV-Cn and WSSV-Tn isolated genome, respectively [13,22]. Most of these ORFs encoded polypeptides that noticeably lacked sequence homology to known proteins. This limitation made it difficult for us to establish the functions of the WSSV proteins. As reported, *wsv023* was expressed at the early stage of WSSV infection, suggesting that it might be indispensable for WSSV [21]. In our previous study, *wsv023* was found to be upregulated by activating transcription factor 4, which is the basic region leucine zipper domain of the transcription factor of *L. vannamei* unfolded protein response [20], whereas its role in WSSV infection was still unclear. The wsv023 sequence was found to be similar to the N-terminal end of GCP3, which had 35.2% identity to HsGCP3 and 31.3% identity to LvGCP3. Surprisingly, wsv023 had higher identity with HsGCP3 than LvGCP3, and LvGCP2 had higher identity with mammal GCP2s than most insect GCP2s, except for *Zootermopsis nevadensis* GCP2 (ZnGCP2). These evidences suggest that wsv023 might be related to *L. vannamei* γ-TuSC.

GCP3 interacted with GCP2 through its N-terminal, interacted with γ-tubulins through its C-terminal, and formed the γ-TuSC, which is involved in microtubule growth initialization [23]. For some viruses, microtubules play a critical role in their infection, such as viral genome transport and envelope assembly; for other viruses, microtubules contribute to the immunologic process of host cells, such as phagocytosis vesicle transport, and microtubule formation should be depressed to support virus infection [24–26]. Thus, viruses develop different strategies to modify the microtubule organization of host cells to ensure successful infection. The most common strategy is to interact with a microtubule or its relevant complexes. Elongation protein zeta 1 promotes neurite extension through its interaction with microtubules; agnoprotein facilitates human polyomavirus John Cunningham virus propagation by inducing the dissociation of FEZ1 from microtubules [27]. Reovirus core protein μ2 determines the filamentous morphology of viral inclusion bodies by interacting with and stabilizing microtubules [28]. Furthermore, vaccinia virus proteins A10 L and L4R with MAP-like properties mediate the direct binding of viral cores to microtubules and disrupt microtubule organization and centrosome function [29]. In the present study, we demonstrated that the WSSV protein wsv023 interacts with LvGCP2, thereby indicating that it might influence microtubule formation in *L. vannamei* cells.

GCP2, GCP3 and two copies of γ-tubulins form γ-TuSC, which possess microtubule-nucleating activity and plays an important role in microtubule initiation [3]. Depletion of either GCP2 or GCP3 from *D. melanogaster* S2 cells eliminates the localization of γ-tubulin at the centrosomes and spindles; this elimination results in gross abnormalities in microtubule organization [30,31]. The formation of microtubules was inhibited by the overexpression of wsv023 in 293T cells, which suggests that the interaction between wsv023 and GCP2 could decrease microtubule-nucleating activity. The wsv023 product is similar to the N-terminal part of GCP3, which interacts with γ-tubulin at its C-terminal end. The wsv023–GCP2 complex prevented the formation of functional γ-TuSC but the effect of this interaction needs further investigation.

Previous reports have suggested that WSSV macromolecule transportation or morphological changes use the cytoskeletal framework of host cells. For example, studies have shown that WSSV infection upregulates the expression of *tubulin α-1 chain*, *tubulin β-1 chain* and *microtubule-actin cross-linking factor 1*, which is indicative of the relationship of WSSV infection and microtubule organization [14–16]. However, direct experimental evidence supporting these concepts is warranted. Flow cytometry analysis indicated that WSSV infection inhibits microtubule formation. In addition, knocked-down expression of *wsv023* resulted in a decrease in WSSV copy number in the muscles of WSSV-infected *L. vannamei*, as well as in its cumulative mortality. These results provide evidences that wsv023-driven modifications in microtubule organization are also essential to WSSV infection.

**Figure 6.**
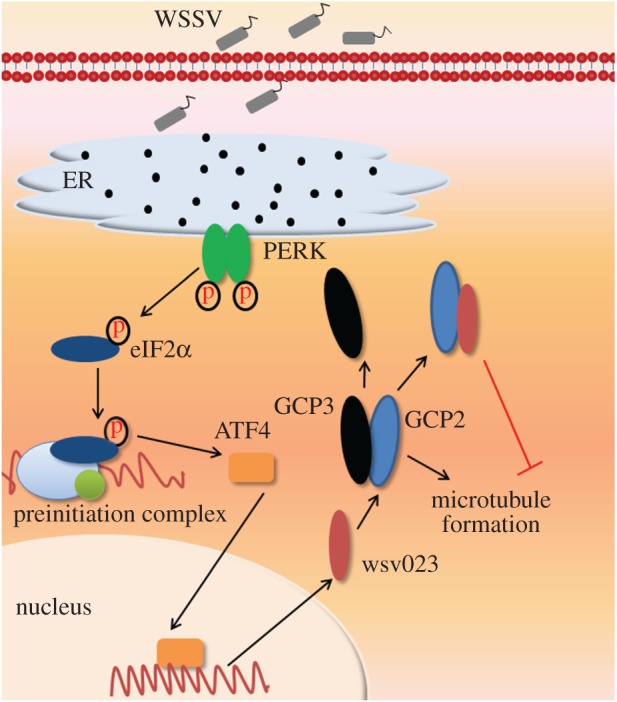
Illustration of wsv023 influence on the microtubule formation in WSSV-infected cells. WSSV infection activated the PERK-eIF2α pathway, which enhanced the expression of *LvATF4*. Then LvATF4 increased the expression of *wsv023* and WSSV copies. Moreover, wsv023 interacted with LvGCP2, which should interact with LvGCP3 under natural conditions, and inhibit microtubule formation of *L. vannamei* cells.

In conclusion, this study indicates that wsv023 interacts with LvGCP2, and might disturb microtubule formation in shrimp haemocyte, promoting WSSV infection (figure 6). Further study will focus on how WSSV may benefit from inhibiting microtubule organization.

## Supplementary Material

Supplemental figure 1

## Supplementary Material

Supplemental figure 2

## Supplementary Material

Supplemental figure 3

## Supplementary Material

Supplemental table 1

## Data accessibility

The nucleotide sequences of LvGCP2 and LvGCP3 had been submitted to NCBI database, and with GenBank accession nos. KR733678 and KR733679, respectively. Other data are available within the electronic supplementary material.
