# Supplementary material for: Wsv023 interacted with Litopenaeus vannamei γ-tubulin complex associated proteins 2, and decreased the formation of microtubules
Source: R Soc Open Sci. 2017 Apr 26;4(4):160379. doi: 10.1098/rsos.160379 (PMC5414238; doi:10.1098/rsos.160379)
Supplement: Supplemental figure 1 [file rsos160379supp1.docx]

**
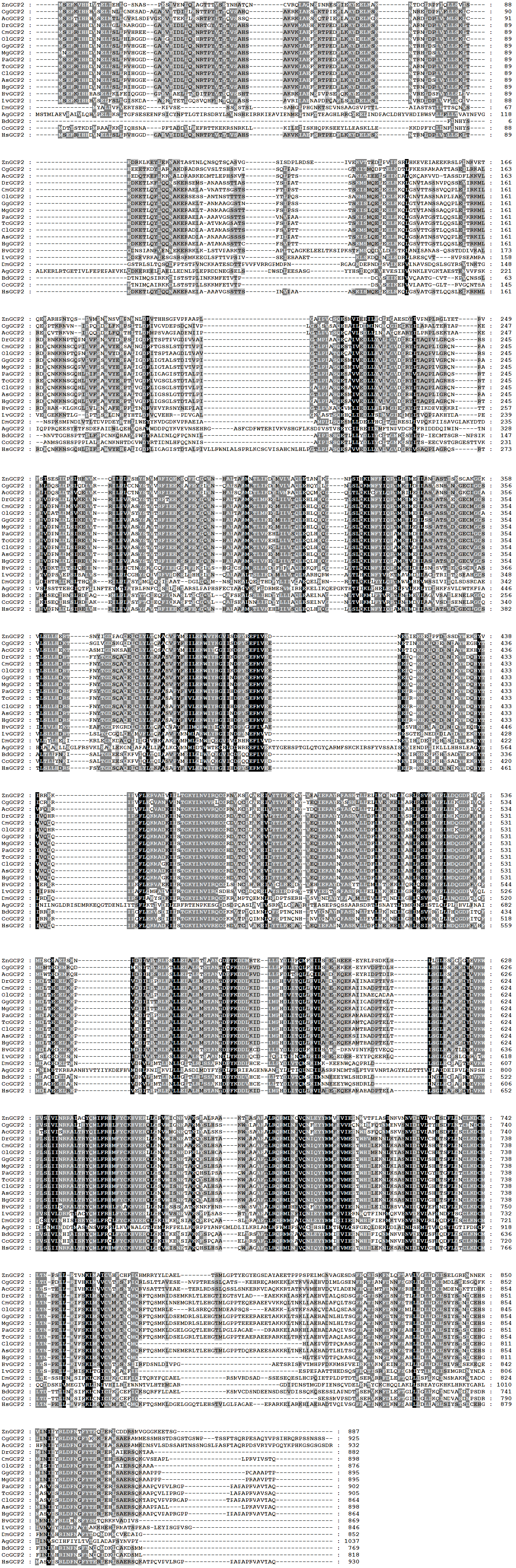
**

**Supplemental figure 1. Multiple sequence alignment of the GCP2 proteins.** This sequces used in this study were AcGCP2, *Aplysia californica* GCP2 (GenBank accession No. XP_005091505); AgGCP2, A*nopheles gambiae str. PEST* GCP2 (GenBank accession No. EAA01681); AsGCP2, *Alligator sinensis* GCP2 (GenBank accession No. XP_006032987); BdGCP2, *Bactrocera dorsalis* GCP2 (GenBank accession No. JAC48755); CcGCP2, *Ceratitis capitata* GCP2 (GenBank accession No. JAB97620); CgGCP2, *Crassostrea gigas* GCP2 (GenBank accession No. XP_011429811); CmGCP2, *Chelonia mydas* GCP2 (GenBank accession No. XP_007057548); ClGCP2, *Chinchilla lanigera* GCP2 (GenBank accession No. XP_005402115); DmGCP2, D*rosophila melanogaster* GCP2 (GenBank accession No. NP_728264); DrGCP2, *Danio rerio* GCP2 (GenBank accession No. NP_956416); GgGCP2, *Gallus gallus* GCP2 (GenBank accession No. NP_001006496); HgGCP2, *Heterocephalus glaber* GCP2 (GenBank accession No. XP_004881481); HsGCP2, *Homo sapiens* GCP2 (GenBank accession No. NP_001243546); HvGCP2, *Hydra vulgaris* GCP2 (GenBank accession No. XP_002154858); LvGCP2, *Litopenaeus vannamei* GCP2 (GenBank accession No. KR733678); MgGCP2, *Meleagris gallopavo* GCP2 (GenBank accession No. XP_010712460); OlGCP2, *Oryzias latipes* GCP2 (GenBank accession No. XP_004080710); PaGCP2, *Pongo abelii* GCP2 (GenBank accession No. XP_009244231) ; TcGCP2, *Tupaia chinensis* GCP2 (GenBank accession No. XP_006147008); ZnGCP2, *Zootermopsis nevadensis* GCP2 (GenBank accession No. KDR14696).
