# Supplementary material for: Wsv023 interacted with Litopenaeus vannamei γ-tubulin complex associated proteins 2, and decreased the formation of microtubules
Source: R Soc Open Sci. 2017 Apr 26;4(4):160379. doi: 10.1098/rsos.160379 (PMC5414238; doi:10.1098/rsos.160379)
Supplement: Supplemental figure 2 [file rsos160379supp2.docx]

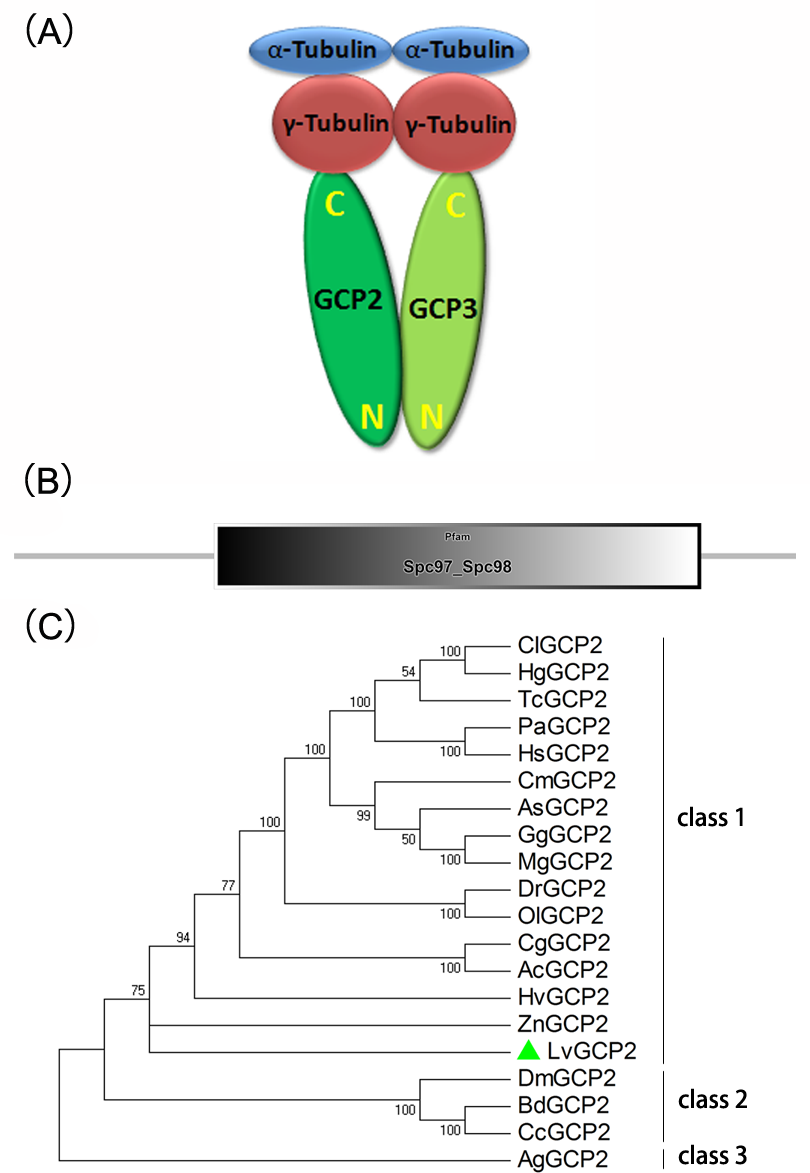


**Supplemental figure 2. Schematic representation of the structural domains of LvGCP2 and phylogenetic analysis of the GCP2 proteins.** . (A) Schematic representation of the structural domains of LvGCP2; (B) Phylogenetic tree of GCP2 proteins frominvertebrates and vertebrates. The tree was constructed by a neighbor-joining algorithm using the Mega 6.0 program based on the multiple sequence alignment by ClusterX v1.83. The LvGCP2 protein was marked by a green triangle.
