# Supplementary material for: Wsv023 interacted with Litopenaeus vannamei γ-tubulin complex associated proteins 2, and decreased the formation of microtubules
Source: R Soc Open Sci. 2017 Apr 26;4(4):160379. doi: 10.1098/rsos.160379 (PMC5414238; doi:10.1098/rsos.160379)
Supplement: Supplemental figure 3 [file rsos160379supp3.docx]

**
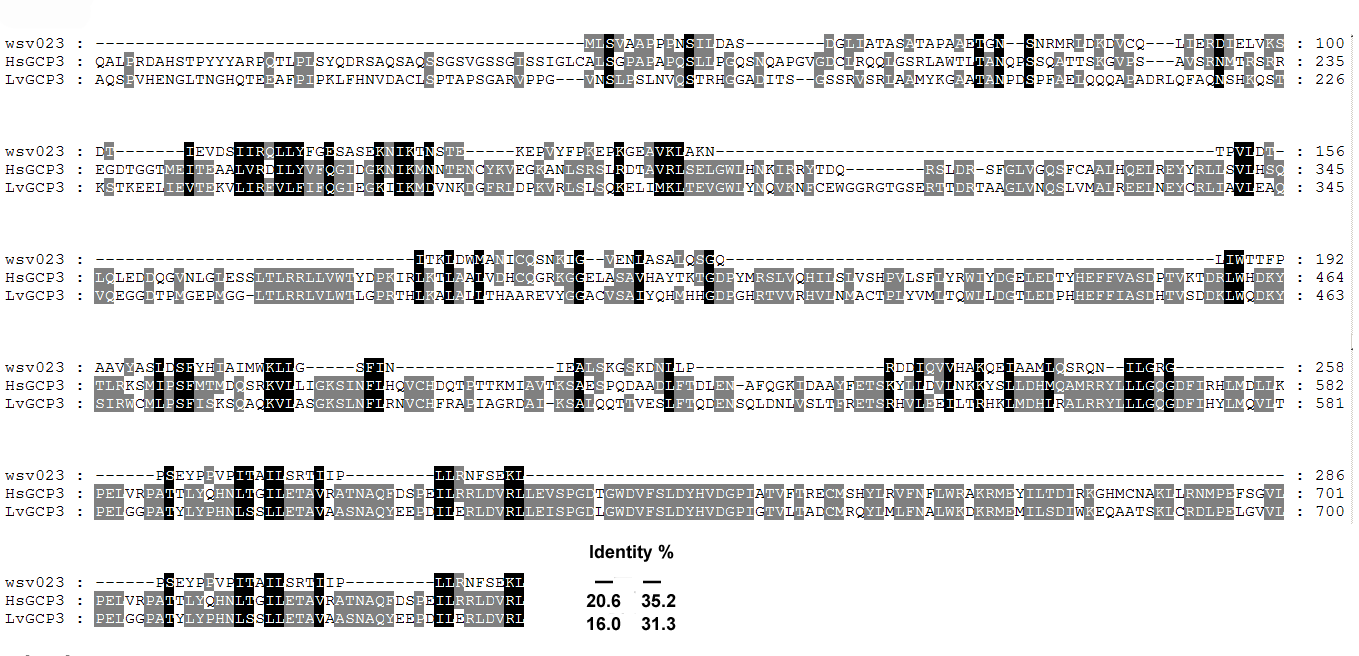
**

**Supplemental figure 3. Similarity analysis of wsv023.** similarity analysis of wsv023 with *Litopenaeus vannamei* GCP3 and *Homo sapiens* GCP3.
