# Supplementary material for: Wsv023 interacted with Litopenaeus vannamei γ-tubulin complex associated proteins 2, and decreased the formation of microtubules
Source: R Soc Open Sci. 2017 Apr 26;4(4):160379. doi: 10.1098/rsos.160379 (PMC5414238; doi:10.1098/rsos.160379)
Supplement: Supplemental table 1 [file rsos160379supp4.docx]

**Supplemental table1. WSSV copy number in muscle of shrimp injected with PBS, dseGFP or dswsv023, plus WSSV infection**

|  | PBS+WSSV^a^ | dseGFP+WSSV^a^ | dswsv023+WSSV^a^ |
| --- | --- | --- | --- |
| 12 h | 4.82 | 4.97 | 5.14 |
| 24 h | 6.16 | 5.52 | 5.30 |
| 48 h | 6.50 | 6.06 | 5.15 |
| 72 h | 7.09 | 6.80 | 6.04 |

^a^ Average value of log10 WSSV copy number per 1 g muscle.
